# The tyrosyl-tRNA synthetase like gene located in the tyramine biosynthesis cluster of *Enterococcus durans *is transcriptionally regulated by tyrosine concentration and extracellular pH

**DOI:** 10.1186/1471-2180-12-23

**Published:** 2012-02-14

**Authors:** Daniel M Linares, Maria Fernández, Beatriz Del-Río, Victor Ladero, Maria Cruz Martin, Miguel A Alvarez

**Affiliations:** 1 Instituto de Productos Lácteos de Asturias (IPLA-CSIC), Carretera de Infiesto s/n, 33300 Villaviciosa, Spain

## Abstract

**Background:**

The tyramine producer *Enterococcus durans *IPLA655 contains all the necessary genes for tyramine biosynthesis, grouped in the TDC cluster. This cluster includes *tyrS*, an aminoacyl-tRNA synthetase like gene.

**Results:**

This work shows that *tyrS *was maximally transcribed in absence of tyrosine at acidic pH, showing a greater than 10-fold induction in mRNA levels over levels occurring in presence of tyrosine. Mapping of the *tyrS *transcriptional start site revealed an unusually long untranslated leader region of 322 bp, which displays the typical features of the T box transcriptional attenuation mechanism. The tyrosine concentration regulation of *tyrS *was found to be mediated by a transcription antitermination system, whereas the specific induction at acidic pH was regulated at transcription initiation level.

**Conclusions:**

The expression of the *tyrS *gene present in the TDC cluster of *E. durans *is transcriptionally regulated by tyrosine concentration and extracelular pH. The regulation is mediated by both an antitermination system and the promoter itself.

## Background

Biogenic amines (BA) are low molecular weight organic bases present in a wide range of food products where they become organoleptically undesirable [1]. It is also worth noting that several toxicological problems resulting from the ingestion of food containing large amounts of BA have been described [2]. Although there is no specific legislation regarding BA content in many food products, it is generally assumed that they should not be allowed to accumulate [3-5].

Fermented foods are likely to contain high levels of BA, mainly due to the decarboxylase activity of some lactic acid bacteria (LAB). BA are produced by the decarboxylation of a precursor amino acid by the enzymatic action of an amino acid decarboxylase [6,7]. In these foods, the main BA are tyramine, histamine, cadaverine and putrescine, which are produced by decarboxylation of tyrosine, histidine, lysine and ornithine, respectively [8].

The presence of the genes encoding the amino acid decarboxylase and the amino acid-amine antiporter is a general feature observed in all the gene clusters involved in the biosynthesis of tyramine, histamine, putrescine and cadaverine [9-12]. We have found an open reading frame coding for a protein of 418 amino acids with a molar mass of 47.38 kDa located next to the tyrosine decarboxylase (*tdcA*) and the tyrosine-tyramine antiporter (*tyrP*) genes of *Enterococcus durans *IPLA655. The predicted amino acid sequence shares strong similarity to the tyrosyl-tRNA synthetase genes (*tyrS*) of gram positive bacteria. The aminoacyl-tRNA synthetases catalyze the covalent attachment of amino acids to their cognate tRNAs, a crucial reaction for the accuracy of protein synthesis. These enzymes are encoded by genes regulated strictly by antitermination systems; when the corresponding amino acid, tyrosine in this case, is at low concentration, it is not linked to the tRNA, and this uncharged tRNA interact with the antiterminator located between the promoter and the start codon, stabilizing it and allowing transcription. By contrast, when tyrosine is at high concentration, it is linked to the corresponding tRNA (charged tRNA) that cannot stabilize the antiterminator, and consequently the transcription stops [13]. Genes with high similarity to those encoding the corresponding aminoacyl-tRNA-synthetases are generally associated to the genes involved in BA biosynthesis in other species, in the case of tyramine (*Enterococcus faecalis *JH2-2 [14], *Enterococcus faecalis *V583 [15], *Enterococcus faecium *MV5 (GenBank HM921050), *Enterococcus hirae *(GenBank AY303667), *Carnobacterium divergens *508 [16]), *Lactobacillus brevis *IOEB9809 [17], *Lactobacillus brevis *ATCC367 (GenBank NC_008497), and the histamine (*Lactobacillus buchneri *B301 [10], *Lactobacillus hilgardii *0006 [18]).

The number of *tyrS *genes is heterogeneous within the genera *Enterococcus*. Some tyramine-producing species as *E. faecium *have a unique *tyrS*, whereas *E. faecalis *has two different *tyrS *genes [15]. So far, this aspect remains unknown for *E. durans *(no genomic data are available). Concerning the two different genes encoding tyrosyl-tRNA-sinthetases in *E. faecalis *V583, *tyrS-2 *is homologous to the *tyrS *of other bacteria with a unique *tyrS*, whereas *tyrS-1 *is located in the tyramine cluster. Database search revealed that *tyrS-1 *has higher similarity (> 80%) to other *tyrS *genes associated to tyramine biosynthesis clusters than to its own *tyrS-2 *gene located elsewhere in the genome (52%). The presence of two *tyrS *genes in the genome of a tyramine producing strain suggests that one (*tyrS-2*) would be implicated in protein biosynthesis, whereas the one linked to TDC cluster (*tyrS-1) *could be a sensor of the intracellular tyrosine pool to regulate tyrosine decarboxylation [9]. In addition, phylogenetic analyses of TyrS proteins associated to tyramine clusters, supported the hypothesis that these proteins made tight clusters and were clearly separated from their relatives encoded elsewhere in bacterial genomes. These results suggested a co-evolution of *tyrS *together with *tdcA *and *tyrP*. This fact prompted us to exhaustively investigate the transcriptional regulation mechanism of this gene and its putative role on the regulation of the tyramine operon.

## Results

### *tyrS *expression depends on the tyrosine concentration and extracellular pH

**Figure 1:**
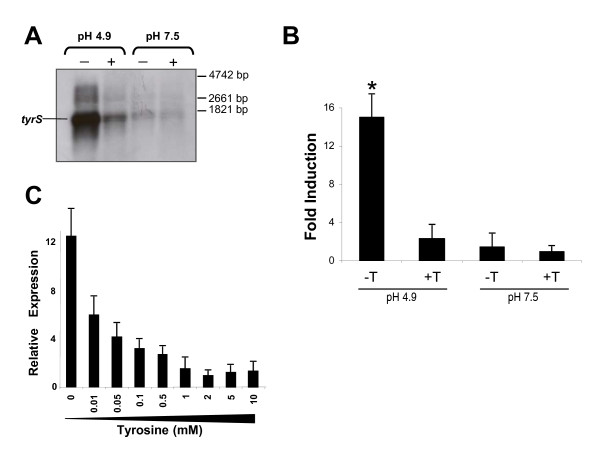
**Transcriptional analysis of the *tyrS *gene**. **A: ***Northern blot *analysis with a *tyrS*-specific probe and **B: **RT-qPCR quantification of *tyrS *mRNA levels in presence (+Y) and absence (**-**Y) of 10 mM tyrosine and different pH conditions (pH 4.9 and pH 7.5). The asterisk indicates statistically significant difference (*p *≤ 0.005; Student's *t*-test) in comparison to the other conditions. **C: **Effect of different tyrosine concentrations (0, 0.01, 0.05, 0.1, 0.5, 1, 2, 5 and 10 mM) on *tyrS *expression at pH 4.9

RNA from *E. durans *IPLA655 cultures grown in presence (10 mM) or absence of tyrosine at two different pH conditions (4.9 and 7.5), was analyzed by Northern blot hybridization with a *tyrS *specific probe (Figure 1A). Very low expression was detected in cells grown at pH 7.5 independently of the presence or absence of tyrosine. Noteworthy, at pH 4.9, an intense band corresponding to a transcript of 1.6 kb was observed. A policystronic mRNA including *tyrS-tdcA *was never detected [19]. The bigger band present in all lines with a low intensity would correspond to unspecific hybridization to the extremely abundant 23S rRNA molecules. This *tyrS *up-regulation was specially enhanced in absence of tyrosine, suggesting that the initiation of transcription or mRNA stability is controlled by pH and tyrosine.

The strength of these environmental conditions on *tyrS *expression was quantified by RT-qPCR. Data in Figure 1B confirmed that *tyrS *is maximally transcribed in absence of tyrosine and at pH 4.9, showing a greater than 10-fold induction in mRNA levels over levels occurring in presence of tyrosine. Even when tyrosine was not added to the media, no induction was detected at pH 7.5. These results confirmed that both conditions (acidic pH and absence of tyrosine) are needed for expression of *tyrS *gene.

Next, we examined whether intermediate tyrosine concentrations have an effect on *tyrS *expression. Therefore, we investigated at optimal pH 4.9, the effect that different tyrosine concentrations in the media (0, 0.01, 0.05, 0.1, 0.5, 1, 2, 5 and 10 mM) exert on gene expression by comparing RT-qPCR results obtained in each condition. As indicated in Figure 1C, *tyrS *expression showed an inverse correlation with the increased tyrosine concentration and exhibited a great sensitivity to very low tyrosine levels, since the maximal expression level was reached in absence of tyrosine and an increase of 0.01 mM tyrosine in the media was enough to reduce this level to the half. Not significant changes in transcription were observed above 2 mM tyrosine, probably because of saturating concentrations of tyrosine. Such concentrations were assayed because tyrosine can reach very high concentrations in some cheeses and even precipitate forming crystals [20].

### Mapping of the *tyrS *transcription initiation site

To map the precise start point of the transcription of *tyrS*, primer extension was performed using RNA samples extracted under optimal conditions of expression (pH 4.9 and absence of tyrosine).

**Figure 2:**
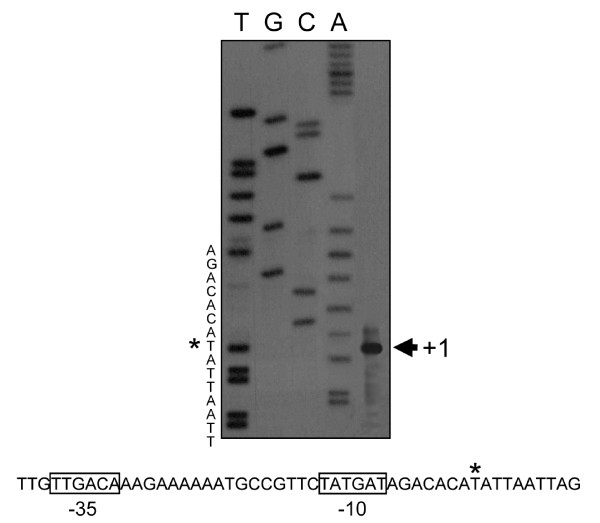
Primer extension identification of transcription start site (*) of *tyrS *and transcriptional regions -10 and -35 (boxes). Lines T, G, C and A correspond to DNA sequence reaction (note that the nucleotide sequences, 5' at the top and 3' at the bottom, represent the complementary DNA strand, so that it may be read directly)

A single band of 322 bp was observed, indicating that the position +1 of the mRNA corresponds to a T residue located 322 nucleotides upstream of the ATG codon (Figure 2). Seven nucleotides upstream this point, it was localized the -10 sequence TATGAT spaced 17 nucleotides downstream of the -35 sequence TTGACA, that nearly matched the consensus sequence for LAB promoters [21]. In a position 9-14 nucleotides upstream the ATG codon of this gene, it was identified the Shine-Dalgarno region (CGGAGG) (bases fitting with the consensus sequences are underlined).

### Structural features of the *tyrS *leader region

**Figure 3:**
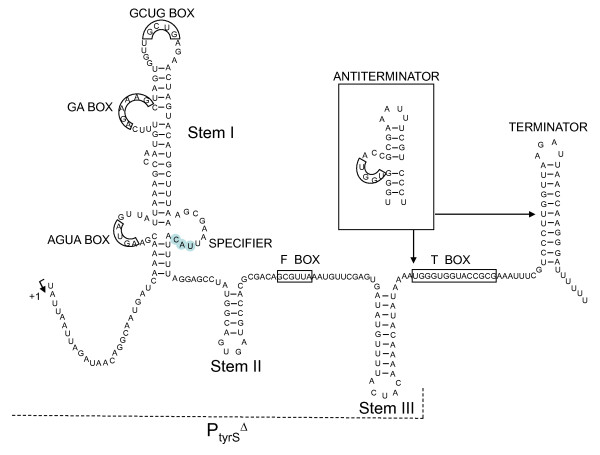
**Primary-sequence and structural model of the *E. durans *IPLA655 *tyrS *mRNA leader region upstream the start of the coding region**. The specifier (UAC), the Tbox sequence, and other highly-conserved motifs typical of genes regulated by tRNA-mediated antitermination appear highlighted in boxes. Sequence between arrows can adopt two alternative mutually exclusive structure conformations: terminator and antiterminator (stabilized by the cognate tRNA in absence of tyrosine). A transcriptional fusion of the *tyrS *promoter and the leader region with a deletion of the TBox-Terminator region (P_tyrS_**^Δ^**) was made (dashed line) to probe the role of the Tbox in the mechanism of tyrosine sensing

In *E. durans *IPLA655, the region upstream of the start codon of *tyrS *showed a 322 bp noncoding sequence that was named the *tyrS *leader region. A hypothetical representation of the secondary structure of the *tyrS *leader region is plotted on Figure 3. This region exhibits the sequence features of the tRNA-mediated antitermination systems described by Grundy et al. [22]. It contains the typical T box sequence UGGGUGGUACCGCG (nucleotides 187-200) (bases fitting with the consensus are underlined), a tyrosine specifier UAC (nucleotides 104-106), and most of the other less conserved boxes (AGUA-I box [AGUA, nucleotides 34-37], GA box [AGAAAG, nucleotides 58-63], GNUG box [GCUG, nucleotides 73-76], and F box [GCGUUA, nucleotides 142-147]). In addition to these conserved sequences, the *tyrS *leader region may be folded into three stem-loop structures (I, II and III) preceding a factor-independent transcriptional terminator/antiterminator. However, the AGUA-II and GAAC boxes that can be found in similar antitermination systems are not present.

### Tyrosine concentration sensing is mediated by an antitermination system

We investigated whether the conserved primary sequence and structural motifs located upstream the start of the coding sequence play a role in the regulation of *tyrS *expression by a transcription antitermination system. For this purpose we compared the amount of mRNA specific of the leader region (mRNA-L) and the amount of mRNA corresponding to the coding part of the gene (mRNA-C) under optimal expression condition (pH 4.9), and in presence or absence of tyrosine. This region-specific transcriptional quantification was performed by RT-qPCR using specific primer pairs for each region (see Methods).

**Figure 4:**
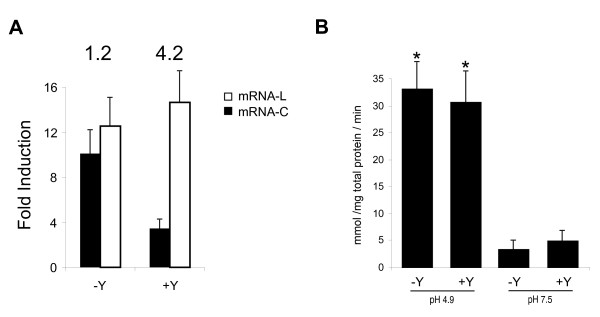
**Regulatory effect of the Tbox on *tyrS *expression**. **A: **Quantification of *tyrS *mRNA-C (*in black*) and mRNA-L (*in white*) levels at pH 4.9 in presence (+Y) and absence (-Y) of 10 mM tyrosine. Numbers above indicate the ratio mRNA-L/mRNA-C in the corresponding condition. **B: **Effect of Tbox deletion on β-Galactosidase activity of P_tyrS_**^Δ^**-*lacZ *fusions at different conditions of pH and presence/absence of 10 mM tyrosine (Y). Data represent the average of three independent experiments. The higher activity observed at pH 4.9 (asterisks) was statistically significant (*p *< 0.005; Student's *t*-test) in comparison to that at pH 7.5

As shown in Figure 4A, level of mRNA-L was not affected by tyrosine concentration, whereas mRNA-C level did not follow the same profile. In presence of tyrosine, the ratio mRNA-L/mRNA-C was 4.2, whereas this value decreased to 1.2 in absence of tyrosine (optimal conditions for *tyrS *expression). The ratio close to 1 observed in absence of tyrosine indicates no transcription termination and consequently the expression of *tyrS*. The 4.2 ratio indicates higher levels of mRNA-L than mRNA-C, suggesting that the transcription in the presence of tyrosine was under the control of a termination system. These data are coherent with a tyrosine concentration regulation of *tyrS *mediated by a transcription antitermination system.

### Assessment of P_tyrS_^Δ ^activity

The role of the T box in the mechanism of tyrosine sensing by *tyrS *was analyzed using a transcriptional fusion of *lacZ *reporter gene with the *tyrS *promoter and the leader region, but with a deletion of the T box-Terminator motif (P_tyrS_**^Δ^**) (Figure 4B). The *lacZ *activities under the control of P_tyrS_**^Δ ^**at pH 4.9 were similar in the absence (33.8 mmol/mg total protein/min) and presence (31.5 mmol/mg total protein/min) of tyrosine, confirming that tyrosine regulation is located on the T box region. On the other hand, independently of the presence of tyrosine, promoter activities at neutral pH were lower than 5 mmol/mg total protein/min, showing an 8-fold higher strength of P_tyrS_**^Δ ^**under acidic pH than at neutral pH. These data indicate that the induction of *tyrS *expression by pH is transcriptionally regulated by the promoter.

### Putative role of *tyrS *in tyramine cluster

To test the hypothesis that TyrS plays a physiological role on tyramine biosynthesis and/or in the regulation of the related genes (*tdcA *and *tyrP*), *tyrS *was over-expressed under the control of the nisin promoter. In all cases, the concentration of *tyrS *transcripts (assessed by RT-qPCR) was 80-fold over the physiological expression level. The presence of soluble translated TyrS was tested by Anti-HIS immunodetection. An intense band of expected size was observed under induction conditions. Next, we analyzed the *in vivo *effect of the over-expression of *tyrS *in cells grown on the aforementioned conditions, (pH 4.9 in GM17-Y and GM17 + Y media). Negative controls of uninduced cultures were carried in parallel. Under these experimental conditions, level of *tdcA*-specific mRNA (quantified by RT-qPCR) was not affected by the overexpression of *tyrS *(data not shown). In addition, the concentration of tyramine in supernatants was examined by HPLC. Only the expected differences depending on the tyrosine concentration in the media were observed (260 ± 40 μM and 3100 ± 80 μM in GM17-Y and GM17 + Y cultures, respectively), but no significant differences between *tyrS*-induced cultures and the negative control were observed.

## Discussion

The *E. durans *IPLA655 *tyrS *gene present in the tyramine cluster, which encodes a protein with high similarity to tyrosyl-tRNA-synthetases, is controlled by the intracellular concentration of tyrosine in a similar way than aminoacyl-tRNA synthetases are regulated by their substrate amino acid. The analysis of TyrS sequence revealed the typical HIGH and KMSKS domains of class I aminoacyl tRNA synthetases, being the HIGH motif perfectly conserved, and the KMSKS motif is represented by the KFGKT sequence, as in *E. coli *[23], *Bacillus subtilis *[24], and *E. faecalis *[14]. Mapping of the transcriptional start site revealed a long untranslated leader region of 322 bp with a highly conserved set of primary-sequence and secondary structure elements. These elements include three stem-loop structures, a highly conserved 14-bp sequence designated the T box, and a factor-independent transcriptional terminator (Figure 3). These features are also present in other genes of gram positive bacteria, mainly genes encoding aminoacyl-tRNA synthetases, but also amino acid biosynthetic genes and transporters [25-27]. Several studies have revealed a crucial role for conserved leader region motifs in regulation of gene expression at the level of premature termination of transcription [28]. In order to test whether this mechanism regulates the *tyrS *gene of *E. durans *TDC cluster, the levels of mRNA were quantified using specific primers for the leader and coding region of *tyrS*. When *E. durans *was starved for tyrosine, the predominant transcript was a 1.6 kb mRNA fragment, which is the expected size for full-length mRNA (mRNA-C). Interestingly, when tyrosine was present in excess, full-length mRNA was dramatically depleted, whereas the truncated mRNA-L species kept almost constant. Thus, tyrosine had no effect on the total number of mRNA-L molecules but caused a stoichiometric replacement of full-length mRNA by truncated RNA molecules. These data are consistent with the idea that tyrosine controls *tyrS *expression by promoting the premature termination of transcription rather than by inhibiting the initiation of transcription.

Experiments involving transcriptional fusions of the *tyrS *promoter with ß-galactosidase provided evidence for this mechanism. We showed that deletion of the T box-Terminator domain of the leader region originates a complete lost of regulation by tyrosine. Early termination at pH 4.9 in presence of tyrosine observed *in vivo *in the leader *tyrS *mRNA (which shows that this sequence promotes terminator formation specifically in presence of tyrosine) was not observed for the P_tyrS_**^Δ ^**promoter. This effect can be expected because the T box sequence is present in a side bulge of the antiterminator overlapping the terminator-antiterminator structures.

In addition to the tyrosine regulation, transcription of *tyrS *is under strict pH control in *E. durans*, being expressed mostly at acidic growth conditions. The aminoacyl-tRNA synthetases catalyze the covalent attachment of amino acids to their cognate tRNAs. Incorporation of tyrosine to proteins is an essential function for cell life, which should not be restricted to acidic conditions. One explanation for this would be the presence of two different tyrosyl-tRNA synthetases, one of which would be induced under stress conditions (acidic pH and extremely low tyrosine concentration). In general, there is only one aminoacyl-tRNA-synthetase for each amino acid in most bacteria, however, several exceptions are known. Indeed, two very similar lysyl-tRNA synthetases, *lysS *(constitutive) and *lysU *(heat inducible) have been described in *Escherichia coli *[29]. In gram-positives, in addition to the aforementioned case of the two *tyrS *of *E. faecalis*, there are two distinct histidyl-tRNA synthetase genes in *Lactococcus lactis *[30], and two tyrosyl-tRNA synthetase genes (*tyrS *and *tyrZ*) and two threonyl-tRNA synthetase genes (*thrS *and *thrZ*) in *Bacillus subtilis *[31,32]. In this last case, the normally silent *thrZ *gene is induced during threonine starvation or by reducing the intracellular concentration of ThrS, which is the housekeeping threonyl-tRNA synthetase sufficient for normal cell growth [33].

**Figure 5:**
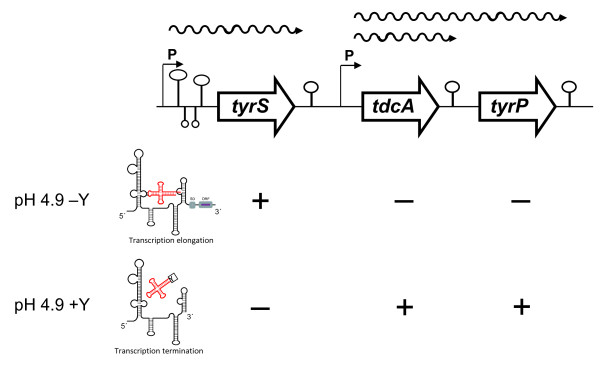
**Genetic organization and transcriptional profile of the TDC cluster in *E. durans *IPLA655**. Promoters (P) and termination regions are indicated. The different mRNA are represented by wavy lines. Numbers indicate the size of the corresponding gene in base pairs (bp). Regulation of the genes by tyrosine and pH is indicated below. Acidic pH is required for optimal expression of the three genes. In addition, *tdcA *and *tyrP *are positively regulated by 10 mM tyrosine (+Y), whereas expression of *tyrS *is negatively affected by tyrosine (+: positively regulated; -: negatively regulated; *tyrS*: tyrosyl-tRNA synthetase; *tdcA*: tyrosine decarboxylase; *tyrP*: tyrosine-tyramine antiporter). Structures upstream *tyrS *represent the stems I, II, III and terminator of the leader region. The terminator/antiterminator mechanism that regulates the *tyrS *gene is also indicated: readthrough of the leader region is induced by limitation of tyrosine. Uncharged ^tyr^tRNA stabilize formation of antiterminator structure in the mRNA, which prevents terminator formation (SD: Shine-Dalgarno; ORF: open reading frame of *tyrS*)

The location of genes encoding an aminoacyl-tRNA-synthetase associated to the gene clusters involved in tyramine and histamine biosynthesis is a general feature [9,10,14,16-18,34]. One of the reasons to study the expression of *tyrS *in *E. durans *is to find out whether this protein could have a role on the genetic regulation of the tyramine cluster, being activated under limiting levels of tyrosine to prevent massive decarboxylation of this amino acid, ensuring its availability for protein synthesis. Consistent with this idea would be 1) the common location of genes encoding aminoacyl-tRNA sinthetases next to the operon of decarboxylation (BA-biosynthesis) of the corresponding aminoacid [9,10,14,16-18,34], 2) the expression of this gene only under acidic pH, which is the condition regulating positively the biosynthesis and accumulation of tyramine [19,35] and 3) the fact that *tyrS *and the genes of the tyramine biosynthesis pathway (*tdcA *and *tyrP*) require opposite conditions of tyrosine concentration for optimal expression (Figure 5) [19]. Altogether, these data raise the question whether TyrS could act as a negative regulator. However, overexpression of *tyrS *on multicopy plasmid during growth of the *E. durans *strain carrying the wild-type allele had no observable effect on the expression profile of the decarboxylating gene *tdcA *or on the tyramine concentration observed in the supernatant.

**Figure 6:**
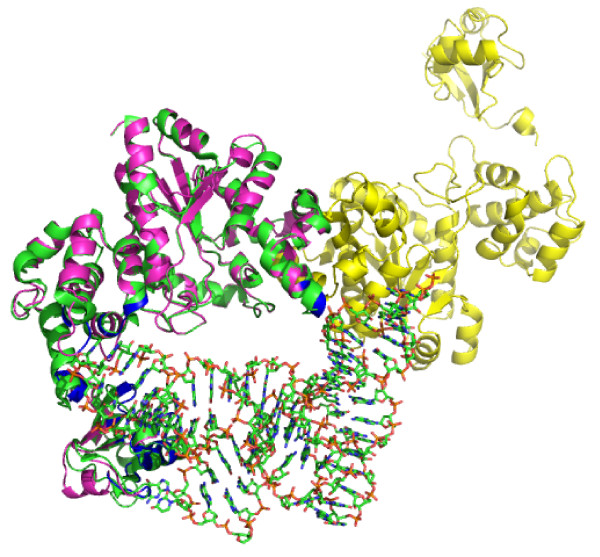
**TyrS structural model achieved using Swiss-Pdb Viewer v. 4.04 software and structure superposition onto the highly similar *Thermus terhmophilus *tyrosyl-tRNA synthetase**. (Protein Data Bank: 1H3E). 1H3E is shown in green, and TyrS model is shown in magenta and yellow. Analysis of the two aligned structures indicates that all of the DNA/RNA binding sites are in regions that interact with tRNA in the 1H3E structure (shown in blue).

Computational three-dimensional modelling of *E. durans *TyrS protein revealed nucleic acids-binding domains that might suggest a role as transcriptional regulator. However, the same domains have been identified in the highly similar TyrS structure of *Thermus thermophilus *(Protein Data Bank: 1H3E), and predicted to interact with tRNA (Figure 6). This data is consistent with the electrophoretic mobility shift (EMSA) assays carried to test TyrS binding to the promoters of the TDC operon. Under the wide range of conditions studied (different pH, salt concentration, presence or absence of tyrosine...) no specific binding of TyrS was observed (data not shown). These data, together with the finding of tyramine clusters without a *tyrS *gene in *Tetragenococcus halophilus *(GenBank AB059363) and histamine biosynthesis clusters without a *hisS *gene [36], would suggest a non critical biological function of these genes in the modulation of the contiguous decarboxylation operon. In any case, it can not be discarded that *tyrS *could exert a post-transcriptional regulation of tyramine biosynthesis. In fact, both enzymes -TyrS and TdcA- share tyrosine as substrate.

Consequently, two are the possibilities that can be considered: i) there are two *tyrS *genes in *E. durans *-as described for *E. faecalis- *and the one ligated to TDC would be a stress-related gene to ensure sufficient charged ^Tyr^tRNA for protein biosynthesis in those conditions that tyrosine is being decarboxylated, or ii) this is the unique *tyrS *gene and the low expression levels observed under neutral pH conditions are enough to assure protein synthesis for general metabolism and the increased expression at acidic pH would guarantee protein biosynthesis when tyrosine is being decarboxylated. The presence of a second *tyrS *gene was investigated by Southern hybridizations of *E. durans *IPLA655 total DNA using as probes the *tyrS *gene of its own tyramine cluster and also *tyrS2 *from *E. faecalis *V583 (the one that is not linked to the tyramine cluster), and no additional *tyrS *genes were found (data not shown). Consistent with this would be the impossibility to knockout the *tyrS *gene, which would support the hypothesis of a single *tyrS*, therefore essential. Then, the most plausible model is the presence of a unique *tyrS *which exhibits a severe induction under acidic pH and low expression levels under neutral pH. It is important to highlight that the transcription quantification is relative, all the expression values were standardized to the reference condition, in this case pH 7.5 (Figure 1B). In fact, the signal observed by Northern blot (Figure 1A) is not zero in any of tested conditions. The *tyrS *basal expression that takes place at neutral pH would be enough to assure protein synthesis.

## Conclusions

In this paper, we provide evidence that transcription of the *E. durans *IPLA655 *tyrS *gene is controlled at two levels, initiation and elongation. The initiation of transcription was shown to be enhanced in acidic environments, whereas elongation of transcription is subject to a tyrosine-dependent attenuation mechanism related to the T box system. This dual mechanism has thus never been described, since previously reported genes induced by tRNA-mediated antitermination, were not found to be pH dependent.

## Methods

### Bacterial strains and growth conditions

The bacterial strains used in this study are listed in Table 1. *Escherichia coli *TOP10 (Invitrogen A/S, Taastrup, Denmark) and *Lactococcus lactis *NZ9000 were used for plasmid propagation. *E. coli *cells were grown in aeration in Luria-Bertani medium at 37°C. *L. lactis *and *E. durans *strains were grown at 30°C on M17 medium (Oxoid, Hampshire, United Kingdom) supplemented with 0.5% glucose (GM17) containing 10 mM tyrosine (GM17 + Y), or on GM17 without tyrosine (GM17-Y). The media was previously adjusted to the corresponding pH condition (pH 4.9 or pH 7.5). When needed, erythromycin (5 μg ml^-1 ^for *E. durans *and *L. lactis*), ampicillin (100 μg ml^-1 ^for *E. coli*) or chloramphenicol (5 μg ml^-1 ^for *E. durans *and *L. lactis*) were added to the culture media.

**Table 1:** Strains and plasmids used in this study

Strain/Plasmid	Characteristics *	Source
**STRAINS**		
*E. coli *TOP10		Invitrogen
*L. lactis *NZ9000	Plasmid-free strain	[37,38]
*E. durans *IPLA655	Isolated from artisanal cheese. Tyramine producer	IPLA Collection
**PLASMIDS**		
pUC18	Amp^R^	[39]
pNZ9530	Ery^R^, *nisR-nisK*	[40]
pILORI4	Ery^R^, *lacZ *promoterless gene	[41]
pNZcLIC	Cm^R^, expression vector	[42]
pDA12	Amp^R^, pUC18 with P_tyrS _cloned in *Sma*I	This work
pDA15	Amp^R^, pUC18 with P_tyrS_**^Δ ^**cloned in *Sma*I	This work
pDA16	Ery^R^, pILORI4 including P_tyrS_**^Δ ^***-lacZ *fusion	This work
pNZcTyrS	Cm^R^, pNZcLIC including *tyrS*	This work

* Amp^R^, ampicillin resistant; Ery^R^, erythromycin resistant; Cm^R^, chloramphenicol resistant

### DNA manipulation procedures

Procedures for DNA manipulation, transformation of *E. coli *cells, and recombinant techniques used were essentially those described by Sambrook et al. [43]. Large-scale isolation of *E. coli *plasmids for nucleotide sequence analysis was performed with the Plasmid Midi Kit (Qiagen Ltd., Crawley, United Kingdom) according to the manufacturer's instructions. Constructions for *E. durans *were achieved using *L. lactis *NZ9000 as intermediate host. Plasmid and chromosomal DNA of *E. durans *and *L. lactis *were isolated and transformed as described previously [44]. All enzymes for DNA technology were used according to the manufacturer's specifications. DNA hybridizations were performed using the non-radioactive DNA Labelling and Detection Kit (Roche Molecular Biochemicals) following the manufacturer's instructions.

### RNA manipulation and northern blot analysis of *tyrS *transcripts

Total RNA was isolated from cells of *E. durans *IPLA655 grown in GM17-Y and GM17 + Y at pH 4.9 and pH 7.5 to exponential phase (optical density at 600 nm [OD600] of 0.6). Purified RNAs were resuspended in DEPC 0.1% (diethyl pyrocarbonate) treated water, and total concentration and yield were determined by UV spectrophotometry by measuring absorbance at 260 nm using a BioPhotometer (Eppendorf, NY).

After extraction, RNA samples were treated with DNase (Fermentas, Vilnius, Lithuania), as described by the manufacturer, to eliminate any genomic contaminations. 20 μg of each sample were subjected to electrophoresis through a 1.5% agarose gel containing 5% formaldehyde and 1X MOPS buffer [20 mM 3-N-morfolino-propanesulfonic acid (MOPS), 1 mM EDTA, 5 mM sodium acetate; pH 7.0]. Transfers and hybridizations were performed as described by Sambrook et al. [43]. DNA probes were labeled with [α-^32^P]dATP by nick translation with the DNA polimerase/DNase I (Invitrogen A/S, Taastrup, Denmark). Primers used in the PCR-amplification of the probes are summarized in Table 2.

**Table 2:** Oligonucleotides used in this study

Primer	Function*	Sequence (5' to 3')
*TDC11* *RT1*	*tyrS *probe amplification (F) *tyrS *probe amplification (R)	TCAATTACAGATCGGTGGGG ACTTACCATCGAATGCATCAAATG
*TRNA2B(2)* *TRNA(P)* *TyrS prom (F)* *tyrS prom (R)*	mRNA-C quantification (F) mRNA-C quantification (R) mRNA-L quantification (F) mRNA-L quantification (R)	CGTAAATTAGAAGGGCCAGAGGCAG GATCAAGCCAGATTGCGCCACCTGCAG AACAGGCAATGATCAAAACGAAGTA CATAGGCTCCTAAAATGTAATTCGC
*TYR2*	P_tyrS _mapping (R)	ACTTCGTTTTGATCATTGCCTG
*TDC123* *TDC130*	P_tyrS_**^Δ ^**cloning and sequencing (F) P_tyrS_**^Δ ^**cloning and sequencing (R)	AAAACTTCCCATATGCATTGTAACG CTGCAGCATTTTATATGTTTTGTAGTAA
*TYS (F)* *TYS(R)*	*tyrS *overexpression (F) *tyrS *overexpression (R)	ATGGGTGGTGGATTTGCTAATATTATCGATGAATTAACTT TTGGAAGTATAAATTTTCATCAACTACTTTGGCCAAAAAG

*F, forward; R, reverse

### Quantification of *tyrS *expression by reverse transcription quantitative PCR (RT-qPCR)

Gene expression analysis was carried out by RT-q PCR on a 7500 Fast Real-Time PCR System (Applied Biosystems, Carlsbad, CA) using SYBR^® ^Green PCR Master Mix (Applied Biosystems, Carlsbad, CA). cDNA samples were synthesized from total RNA using the iScript™ cDNA Synthesis kit (Bio-Rad, Hercules, CA). After dilution of cDNA, 5 μl were added to 20 μl of PCR mixture (12.5 μl of SYBR Green Supermix, 1 μL of each primer at 7 μM, 5.5 μl of RNAse free water). Amplifications were performed with specific primers (Table 2) designed using Primer Express software (Applied Biosystems, Carlsbad, CA), using primers for 16S ribosomal RNA as an internal control to normalize RNA concentration [19]. Cycling settings were those default-established by Applied Biosystems, Carlsbad, CA. For each condition, RT-qPCR analysis was performed on RNA purified from three independently grown cultures.

### Primer extension and DNA sequencing

Total RNA isolated from *E. durans *IPLA655 grown in GM17-Y pH 4.9 was used as template for primer extension. The reaction was done mixing 15 μg of RNA with 1 pmol of oligonucleotide 5'end-labeled with [γ-^32^P]-dATP using T4 polynucleotide kinase (New England Biolabs Inc. Ipswich, MA) and a mixture containing deoxynucleotides triphosphate (1 mM each), RNase inhibitor (Gibco, BRL), and 20 U of Avian Myeloblastosis Virus reverse transcriptase (Promega, Madison, USA). The control sequence used as size standard was determined with the same reverse primer using as template 2 μg of the double-stranded recombinant plasmid pDA12 (Table 1). Each sequence reaction was performed according to the dideoxynucleotide chain termination sequencing method using 5 μCi of [α-^32^P]dCTP and the T7 Sequencing™ Mixes Kit (Pharmacia Biotech Inc. Piscataway) under manufacturer instructions. Finally, the samples were resolved in parallel on a denaturing 8% polyacrylamide gel containing 7 M urea to determine the endpoints of the extension products.

### Construction of P_tyrS_^Δ^-*lacZ *transcriptional fusion

A fragment including the *tyrS *promoter and leader region with a deletion of the T-box-Terminator domain (P_tyrS_**^Δ^**) (Figure 3), was amplified using primers *TDC123 *and *TDC130 *(Table 2) and cloned in the *Sma*I site of pUC18, resulting pDA15 plasmid. A 450 bp *Eco*RI-*Pst*I fragment including P_tyrS_**^Δ ^**was cloned into the low copy number vector pILORI4 [41] for fusion to a promoterless *lacZ *reporter gene, yielding the pDA16 plasmid. All clones were sequenced.

Plasmid pDA16 was firstly constructed in *L. lactis *NZ9000 strain, and subsequently transformed into *E. durans *IPLA655, and *lacZ *activity was tested under specific pH and in presence or absence of tyrosine in the culture media. For promoter assay, a negative control with the pILORI4 plasmid was carried.

### Determination of β-galactosidase activity

In order to determine β-galactosidase activity, cells grown in the corresponding conditions of pH and tyrosine concentration at OD_600 _= 0.6 were harvested, resuspended in 1 ml of 10 mM Na_2_HPO_4_, pH 7.0, and then disrupted with 0.2 g of glass beads (Sigma). After centrifugation at 12000 *g *for 10 min at 4°C, different dilutions of the resulting supernatants (CFE) were immediately assayed in a final volume of 1 ml, which included 750 μl of 10 mM sodium phosphate buffer pH 7.0, 200 μl of CFE and 50 μl of 20 mM *o*-nitrophenilgalactopiranoside (ONPG). The mixture was immediately incubated at 37°C and absorbance was measured (λ = 420 nm). Each condition was assayed independently by triplicate and the values were standardized to protein contents of cell extracts, determined by using the BCA Protein Assay Reagent Kit (Pierce, Rockford, Ill.).

### Overexpression of *tyrS *and immunodetection

The gene encoding for TyrS was amplified using primers TYSF and TYSR (Table 2) and cloned into a pNZcLIC expression vector using the VBEx system [45], yielding the corresponding derivative pNZcTyrS. For detection purposes, a decaHis-tag was added to the C-terminal of the target protein.

*tyrS *expression was carried using the NICE system [46]. The genes encoding *nisR *and *nisK *were introduced in *E. durans *IPLA655 in the low copy number plasmid pNZ9530 [40]. After induction with 2 μg L^-1 ^nisin, expression of the protein was confirmed by Western blotting analysis of cell lysates by 10% SDS-PAGE electrophoresis gels, subsequently electroblotted and immunodetected with an anti-His-tag antibody (Amersham Pharmacia Biotech Inc. Piscataway). Chemiluminescence detection was done using the Western-Light kit (Tropix Inc. Bedford, MA) and quantified using the Fujifilm LAS-3000 imaging system (Fuji Photo Film Co. Ltd; Tokyo).

### Analysis of tyramine by HPLC

The quantitative analysis of tyramine production was undertaken by reverse-phase high performance liquid chromatography (RP-HPLC) using a Waters liquid chromatograph controlled by Millenium 32 Software (Waters, Milford, MA, USA). The samples were prepared by centrifugation at 8,000 × g for 10 min. The resulting supernatants were filtered using Millipore 0.2 μm filters and derivatized using dabsyl chloride, as described by Krause et al. [47]. Separations were performed using a Waters Nova-pack C18 column (150 × 3.9 mm). Usually, 10 μl of the derivatized sample was injected and detection performed at 436 nm. The solvent gradient and detection conditions were similar to those described by Krause et al. [47].

## Authors' contributions

DML designed and performed the experiments, and drafted the manuscript. MF and MAA designed experimental procedures and helped to write the manuscript. All authors read and approved the manuscript.
